# Utilisation of postnatal care among rural women in Nepal

**DOI:** 10.1186/1471-2393-7-19

**Published:** 2007-09-03

**Authors:** Sulochana Dhakal, Glyn N Chapman, Padam P Simkhada, Edwin R van Teijlingen, Jane Stephens, Amalraj E Raja

**Affiliations:** 1 Department of Public Health, University of Aberdeen, Aberdeen, AB25 2ZD, UK; 2 Green Tara Trust, London, UK

## Abstract

**Background:**

Postnatal care is uncommon in Nepal, and where it is available the quality is often poor. Adequate utilisation of postnatal care can help reduce mortality and morbidity among mothers and their babies. Therefore, our study assessed the utilisation of postnatal care at a rural community level.

**Methods:**

A descriptive, cross-sectional study was carried out in two neighbouring villages in early 2006. A total of 150 women who had delivered in the previous 24 months were asked to participate in the study using a semi-structured questionnaire.

**Results:**

The proportion of women who had received postnatal care after delivery was low (34%). Less than one in five women (19%) received care within 48 hours of giving birth. Women in one village had less access to postnatal care than women in the neighbouring one. Lack of awareness was the main barrier to the utilisation of postnatal care.

The woman's own occupation and ethnicity, the number of pregnancies and children and the husband's socio-economic status, occupation and education were significantly associated with the utilisation of postnatal care.

Multivariate analysis showed that wealth as reflected in occupation and having attended antenatal are important factors associated with the uptake of postnatal care. In addition, women experiencing health problems appear strongly motivated to seek postnatal care.

**Conclusion:**

The postnatal care has a low uptake and is often regarded as inadequate in Nepal. This is an important message to both service providers and health-policy makers. Therefore, there is an urgent need to assess the actual quality of postnatal care provided. Also there appears to be a need for awareness-raising programmes highlighting the availability of current postnatal care where this is of sufficient quality.

## Background

Maternal mortality is a major concern of maternal health in developing countries. Maternal mortality in Nepal (in South Asia) is estimated to be 740 per 100,000 live births [1], compared to 12 per 100,000 in a developed country such as the UK [2], and 400 per 100,000 estimated worldwide [3]. The majority of maternal deaths (62%) occurs place soon after birth with postpartum haemorrhage being the major cause of death [4]. The kind of complications following childbirth such as chronic pain, impaired mobility, damage to the reproductive system, genital prolapse and infertility are also more common in developing countries [5].

Postnatal care is one of the most important maternal health-care services for not only prevention of impairment and disabilities but also reduction of maternal mortality. Due to their low social status, poverty and ethnicity women in Nepal have less access to modern health services and more trust in traditional care, for example they are more likely than men to seek help from traditional healers [6]. The utilisation of postnatal care services is low in Nepal; only 21% of new mothers receive it. Similarly, only 17% of mothers received their first postnatal check-up within two days of delivery [7]. Since most maternal deaths occur during delivery and the postpartum period due to complications, the first week after delivery is the most critical time in the postnatal period, with most complications occurring in the first two days. The most common fatal complications are postpartum haemorrhage, sepsis, complication of unsafe abortion, prolonged or obstructed labour and eclampsia [8].

The socio-cultural practices around childbirth such as maternal seclusion after delivery and cultural beliefs in a community play a vital role in non-utilisation of postnatal care services in Nepal [9]. At the same time, decisions about mobility of women and expenditure on health care are controlled by men or older women of the household, which may limit women's search for health care [10].

About 90% of the population lives in rural areas and depends on agriculture. Nepalese women generally have a low social status. According to traditional Nepalese cultural norms, women have to cook and serve food to all other household members before eating themselves. Moreover, they do manual labour as more than 90% of the economically active participants in agriculture are female [11].

Utilisation of postnatal care by women influences both women and children's lives, in terms of reducing repeat pregnancies and increasing effective contraceptive use. Therefore, a proper understanding of the utilisation of health care during the postnatal period can reduce maternal mortality. However, there is no information available about uptake of postnatal care in particular these villages. Considering the circumstances of under-utilisation and lack of resources for maternal health services in Nepal, the need exists to assess the current situation of the utilisation of postnatal care.

Therefore, the objective of our study is to assess the proportion of the utilisation of postnatal care among women in Nepal. The secondary objectives of the study are to assess the association between demographic, socio-economic, care-seeking behaviour during pregnancy and the utilisation of postnatal health care.

## Methods

### Study setting

The study was conducted in two VDCs (Village Development Committee areas) or 'villages' of Kathmandu district, Nepal. These were typical VDCs close to Kathmandu valley which are relatively underdeveloped, but slightly more developed than the average VDCs in Nepal. Village 'D' is 20 km south of Kathmandu Valley. According to the 2001 Census there are a total of 824 households while the total population of the VDC is about 4,500; half of them female [12]. There is a sub-health post (SHP) in each VDC which consists of three regular staff including a Mother and Child Health Worker. In addition, there was a recently established community hospital in VDC 'D'. Some of the wards of VDC 'D' are connected by road to Kathmandu. VDC 'C' is three km from VDC 'D', and also possessed a SHP. The number of households in this second VDC, a total population of just over 4,000 and slightly more than half were women, was similar to that of the first [12]. Both villages contained nine wards.

### Study design and participation

The study used a cross-sectional design. The study population comprised married women in the reproductive age group (15–49 years old), residing in study areas, who had a live baby less than 24 months old.

According to the Census it is estimated that children younger than two comprises 4.23% of the total population [12]. Hence the study population was estimated to be 362 women in the two VDCs combined. Local Female Community Health Volunteers (FCHVs) provided a list of targeted women in each ward. We took a convenience sample of about half of the relevant women. In each village three out of the nine wards were selected at random to yield the required sample size. House-to-house visits were made with the assistance of FCHVs to find eligible women for the study. However, some of the women were missed because they were out during our visit and on a same-day call-back.

The semi-structured questionnaire was designed using the literature on postnatal care, as well as some validated questions from the 2001 Nepal Demographic and Health Survey [7]. The questionnaire elicited information on: the socio-demographics, knowledge, attitudes and practice towards postnatal health care among the target group. Some alterations to the questions were made after the pilot study. A copy of the questionnaire is available on request from the first author. After obtaining informed consent from each woman the first author (female) administered the questionnaire, which took, on average, 20 minutes. Ethical approval for conducting the study was obtained from the Nepal Health Research Council.

### Statistical analysis

Data were entered and analysed using the SPSS version 13.0 for Windows. Pearson's chi-square test was used to find association between nominal categorical factors and postnatal care. A continuity correction test was used to find associations between binary categories of different perceived barrier factors and postnatal care. The Fisher's exact test was used whenever the number of cell in the column was minimal. Chi-square test for trend was used for association of ordinal categorical factors and postnatal care. The Odds Ratio (OR) and its 95% Confidence Interval (CI) were calculated to measure the strength of the association between demographic and socio economic factors, antenatal care uptake on the one hand and on the other postnatal care. Those factors that were significant at 20% level in the univariate analysis were considered for the multivariate analysis. Multivariate logistic regression with backward elimination method was used to find best combination of factors predicting postnatal care. A p-value of less than 0.05 was considered to be statistically significant.

## Results

### Demographic and socio-economic information of respondents

There were 150 women included in the study. The number of study subjects represented from each village 'C' (56%) and village 'D' (44%) was almost equal. The majority of women was 20 to 24 years old and also had their first pregnancy in this age group. Two thirds of women had one or two children and an almost similar proportion was living in families with 5–8 members (Table 1).

**Table 1:** Association between demographic, socio economic factors & postnatal check up

**Factors**	Total (n = 150)	%	**Postnatal check**		OR	95% CI	P-value
						
			Yes (n = 51)	No (n = 99)			
Age group							
< 20	16	11	3	13	1.00		0.210*
20 – 24	76	51	35	36	4.21	0.99–20.52	
25 +	58	39	13	45	1.25	0.27–6.52	
Ethnicity							
Brahmin-Chhetri	25	17	15	10	1.00		0.001
Tamang	89	59	16	71	0.15	0.05–0.44	
Other	36	25	20	13	1.03	0.31–3.38	
Education of women							
Illiterate	73	49	16	54	1.00		0.001*
Primary	37	25	10	27	1.25	0.45–3.42	
Secondary	40	27	25	13	6.49	2.50–17.2	
Occupation of women							
Farmer	107	72	23	80	1.00		0.001
House wife	38	25	25	12	7.25	2.94–18.18	
Other	5	3	3	2	5.22	0.65–48.14	
Education of husband							
Illiterate	21	14	3	17	1.00		0.001*
Primary	55	37	10	43	1.32	0.28–6.92	
Secondary	73	49	38	34	6.33	1.55–29.95	
Occupation of husband							
Farmer	73	49	17	53	1.00		0.005
Formal sector & abroad	17	11	4	12	1.04	0.24–4.15	
Other job	60	40	30	29	3.23	1.43–7.32	
Number family members							
3–4	34	23	12	21	1.00		0.090*
5–8	88	59	29	57	0.89	0.36–2.24	
9+	28	19	10	16	1.09	0.33–3.61	
Number of children							
1 or 2	113	75	47	61	1.00		0.001
3 or more	37	25	4	33	0.16	(0.04–0.51)	
Number of pregnancies							
1	68	45	33	33	1.00		0.001*
2	36	24	9	24	0.38	0.14–1.01	
3 or more	46	31	9	37	0.24	0.09–0.63	
Age at first pregnancy							
≤18	53	35	13	37	1.00		0.140
19+	97	65	38	57	1.89	0.89–4.03	

* Chi-square test for trend

Half of the women were illiterate and of the remaining women, equal numbers had primary education and secondary education or above. Three quarters of the women (72%) were farmers and the rest were housewives (25%) with a negligible proportion having formal sector work (3%).

Half of the husbands were educated to secondary school level and above, and a small proportion was illiterate (14%). Half of the husbands were involved in farming and the others in formal sector work such as in education or the civil service (38%), whilst some worked abroad (4%), e.g. in the Middle East or Malaysia. Other occupations (8%) included students and causal work such as painter or goldsmith.

### Utilisation of postnatal care

**Figure 1:**
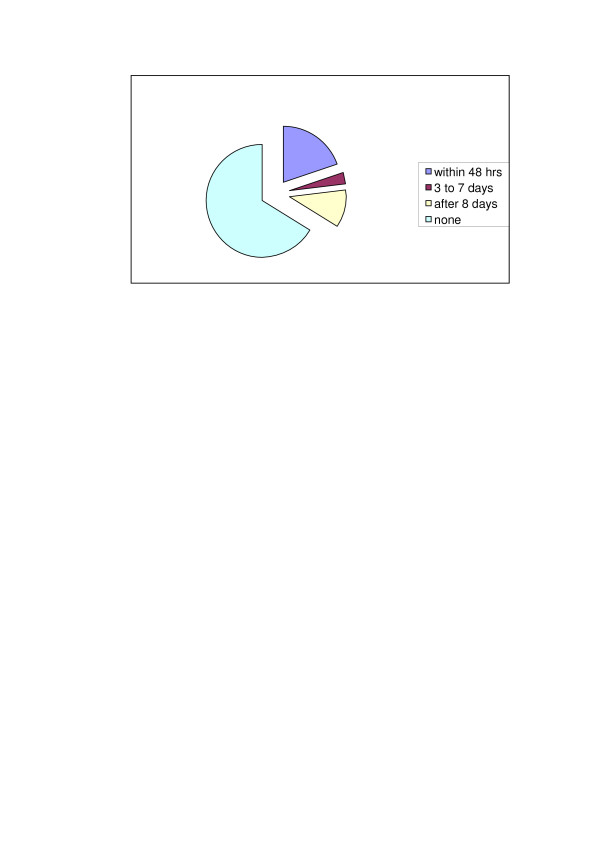
Timing of postnatal health check.

The prevalence of postnatal care was 34% (95% CI = 27% – 42%) within 42 days after delivery (Figure 1), and 19% within 48 hours. Women reported that they had postnatal care from a doctor (65%) rather than a nurse (20%) or other health workers (16%). Similarly, the majority of women (78%) had received their care in hospital.

### Association between demographic/socio-economic factors and postnatal care

An attempt was made to find which socio-demographic factors were associated with postnatal care (Table 1). Postnatal care prevalence was not different among different age groups (p = 0.21). The 'Tamang' ethnic group was less likely to have had postnatal care than Brahmin-chhetri (p = 0.001). The occupation of women was associated with having received postnatal care. Housewives were 7.25 times (95% CI = 2.94–18.18) more likely to have had postnatal care than women who reported farming as their main occupation. It was observed that as education of women increases so did the likelihood of having postnatal health care. Women with secondary school education had 6.49 times (95% CI= 2.5 – 17.2) more chance of receiving postnatal care than illiterate women (p = 0.001). Similarly women with a husband educated to secondary school level (OR = 6.3; 95% CI = 1.55 to 29.95) had a significantly greater chance of having postnatal care than those with an illiterate husband (p = 0.001). The husband's occupation is associated with postnatal care uptake (p = 0.001). Husbands with a formal-sector job such as teaching or civil servant (OR = 3.23; 95% CI = 1.43 – 7.32) were more likely to have wives who attended postnatal care. The number of family members was not associated with postnatal care uptake. However, women with three or more children were less likely to have had postnatal care (OR = 0.16; 95% CI = 0.04–0.51) than a woman with one or two children. Similarly, having had more pregnancies was associated with a lower chance of postnatal care utilisation (p = 0.001). Women who had attended antenatal care were more likely to attend postnatal care (OR = 24.6; 95% CI 3.39 to 500.92). Women who delivered in the hospital were ten times (95% CI= 4.64 to 23.7) more likely to have received postnatal care than the women who delivered at home.

### Perceived barriers to access to postnatal care in the community

All women were asked to give their opinion on barriers to access to postnatal care in their community. Table 2 lists these main barriers: a lack of awareness or no perceived need for postnatal care by women and their families (47%), distance to health facility (39%) and lack of transportation or good roads (23%), lack of money (17%), and lack of skilled health workers in the community (14%).

**Table 2:** Association between perceived barriers for health care & postnatal check up

**Factors**	Total (n = 150)	%	**Postnatal check**		OR	95% CI	P-value
						
			Yes (n = 51)	No (n = 99)			
Health facility far							
Yes	58	39	20	37	0.99	0.50–2.00	1.00
No	92	61	31	57	1.00		
No skilled female worker							
Yes	21	14	11	10	2.31	0.91–5.89	0.12
No	129	86	40	84	1.00		
No money							
Yes	25	17	13	12	2.34	0.97–5.60	0.09
No	125	83	38	82	1.00		
Women & family member not aware							
Yes	70	47	24	45	0. 97	0.48–1.92	1.00
No	80	53	27	49	1.00		
No PNC at local level							
Yes	7	5	4	3	2.58	0.56–12.02	0.40
No	143	95	47	91	1.00		
No time due to home work							
Yes	3	2	1	2	0. 92	0.08–10.39	1.00*
No	147	98	50	92	1.00		
No time due to home work							
Yes	3	1	2	2	0. 92	0.08-10.39	1.000*
No	147	50	92	92	1.00		
No transportation							
Yes	35	23	11	24	0.80	0.36–1.81	0.74
No	115	77	40	70	1.00		
Had antenatal check up							
Yes	117	78	50	63	24.6	3.39–500.9	0.001*
No	33	22	1	31	1.00		
Place of delivery							
Hospital	46	31	32	13	10.49	4.64–23.7	0.001
Home	104	69	19	81	1.00		
Having health problem after delivery							
Yes	15	10	10	4	5.49	1.63–18.53	0.003
No	135	90	41	90	1.00		

* Fisher's exact test

None of the expected barriers: health facilities being far (p = 1.0), having no skilled female worker (p = 0.12), no money (p = 0.89), women and family member not being aware (p = 1.00), no postnatal care at local level (p = 0.40), no time due to need to work at home (p = 1.00) and no transportation (p = 0.74) were statistically associated with postnatal care uptake.

### Health problems during postnatal period and seeking care

All women were asked about health problems they may have had in the postnatal period, only one in ten reported such problems. However, having such health problem was significantly associated with having postnatal care (p = 0.030). The common health problems perceived by women during the postnatal period were weakness (27%), mastitis (27%), vaginal bleeding (20%), fever (13%), vaginal pain (13%) and a prolapsed uterus (7%).

Women, who did not seek care for their health problems during postnatal period, were asked the reason why not. Their main reasons were distance to the health facility followed by inability to walk/travel to such facility.

### Suggestions of women for the improvement of postnatal care

The majority of women recommended that there should be better postnatal care services and skilled health workers in their community, as well as an increased awareness of the availability and importance of postnatal care (Table 3).

**Table 3:** Suggestions given by women for improvement of postnatal care (n = 150)

**Suggestions**	**Number**	%*
Health services on postnatal care in village	77	51
Increased awareness on postnatal care	47	31
Better trained health workers	46	31
More medicine	35	23
More support from family	28	19
Transportation	9	6
Don't know	42	28

* More than one suggestion was allowed per respondent, hence the total is more than 100%.

### Multivariate analysis

The factors which were significantly associated with postnatal health care at 20% level of significant: ethnicity, education of women and their husbands, occupation of women and their husbands, number of children, having had antenatal care, place of delivery, age at pregnancy, having no female health care workers and having a health problem after delivery were entered into multivariate model with backward elimination method to find independent effects on postnatal care. Multivariate logistic regression analysis identified woman and husbands having formal sector jobs or husbands working abroad, having had antenatal care, delivery in hospital and women who reported having health problem after delivery as significantly associated with postnatal care (Table 4).

**Table 4:** Association between demographic, socio economic, antenatal health check & post natal check up using multivariate logistic regression analysis

**Factors**	**Adjusted OR**	95% CI*
Age of women		
< 20	1.00	
20–24	6.74	1.04–43.79
25+	2.55	0.36–18.21
Occupation of women		
Farmer	1.00	
House wife	6.28	2.00–19.69
Other	3.06	0.27–34.64
Occupation of husband		
Farmer	1.00	
Formal sector + worked abroad	0.83	0.27–2.53
Other job	0.15	0.03–0.85
Occupation of husband		
Farmer	1.00	
Formal sector + worked abroad	0.83	0.27–2.53
Other job	0.15	0.03–0.85
Antenatal check up done	11.06	1.16–105.59
Delivery at hospital	10.12	3.40–30.07
Health problem after delivery	17.3	3.36–88.78

* Confidence Interval which excludes 1 is statistically associated with postnatal health check at 5% level of significance

## Discussion

### Utilisation of postnatal care

The National Maternity Care Guidelines in Nepal recommend that postnatal care should be available at the level of the sub-health post and outreach clinics and that all postnatal women should be followed up within two days [13]. A large number of maternal and neonatal deaths occurs during the first 48 hours after delivery. Although the study included both those who delivered outside and within a health facility, the utilisation of postnatal care was found to be low (34%). Moreover, care within 48 hours was found to be infrequent (19%). This is very low compared with the nearly 90% uptake of postnatal services reported in developed countries [14]. Women who delivered at health facilities had more access to postnatal care. However, our study suggests that not all women with a hospital delivery received postnatal care.

The study revealed that women prefer to go hospital for postnatal care rather than to health posts. This might be related to quality of services and trust in health workers. Similarly, more women getting antenatal and delivery care might have a positive influence on the uptake of postnatal care, as the uptake might be associated with health education and counselling received during antenatal visits and the delivery.

Our study found that the number of family members is not associated with postnatal care. These results contradict a previous study in Nepal which indicated that women living in a joint (extended) family are more likely to use maternal health services during the postnatal period than those living in a nuclear family [15]. Women who had fewer children and had received antenatal care were more likely to use postnatal care as found elsewhere [16]. Education of women is a positive factor for utilisation of postnatal care in our study as it is in many other studies conducted in different parts of Asia and Africa [17-19].

Women's occupation showed a significant association with the likely uptake of postnatal care. Education is related to occupation. Educated women are more likely to get a job in the formal sector than in agriculture. Women who are employed not only have better financial status and ability to use quality health services, but also gain empowerment to take part in the decision-making process about healthcare in the family [20-22]. The husband's occupation and education can represent family income as well as social status. Occupation of the husband was also seen as an influential factor for utilisation of postnatal care in the study. The result is supported by a study from Bangladesh which showed a higher use of quality care for postpartum morbidity by wives of businessmen and service workers [21]. Similarly (health care) awareness of women, distance to health facilities, level of education, occupation and the husband's occupation are significantly associated with utilisation of postnatal care in Uganda [22].

### Health problems during the postnatal period and seeking care

Perceived health problems occurring during the postnatal period were found to be low (about 10%) in our study. Possible explanations here are that women and their families were not aware of signs of health problems or that they did not perceive minor illness as a health problem. The most commonly mentioned health problems were weakness, breast infection and vaginal bleeding. Although the great majority of women (87%) had sought help, some had sought care from a traditional healer. This might be due to a lack of: (a) easily accessible health services; (b) skilled health workers; or (c) transport facilities. Although we should not ignore the possibility that some of the traditional healers are effective in the care they provide. Our study results concur with the Nepal Multiple Indicator Surveillance (1997), which showed that ten per cent of mothers had health problems postnatally. Common health problems there were fever/infection excessive vaginal bleeding and weakness. Nearly three quarters women (73%) there had sought help [23].

### Barriers to access to postnatal care

In our study, the main barriers to postnatal care were: lack of awareness among women and their family about the care, distance to health facilities, lack of trained health workers and lack of health facilities in the village. Similarly, women who had low education, the high number of pregnancies and children had less access to postnatal care. Similarly, the Tamang ethnic group, poor, illiterate and farm-working women had less access to postnatal care while the majority of such women were from village C.

The main reason for the non-use of postnatal health services is the lack of awareness or not perceiving a need for it. Lack of awareness might be related to illiteracy and lack of particular health services in accessible area [24].

The availability of services and understanding of the importance of postnatal care are vital to improved uptake of postnatal care [22]. The underutilisation of postnatal care is generally related to unavailability, inaccessibility and the poor quality of health services [25]. Physical accessibility is an important variable for utilisation of postnatal health services [26]. Distance limits women's willingness and ability to seek health care particularly when appropriate transportation is scarce and communications and terrain are difficult.

Women's empowerment factors such as education, exposure to mass media and household autonomy play a major role in the utilisation of maternal health care [17]. Low education and decision-making power of women in Nepal may restrict access to postnatal care. Similarly, lack of female skilled health workers may be another barrier to the uptake of postnatal care, since Nepalese husbands may not be willing to send their wives for medical examination by male doctors [27]. Chronic lack of human resources and geographical maldistribution are barriers in maternal health services in Nepal, especially in the past decade with where these factors suffered during the Maoist insurgence. Moreover, very poor roads and a lack of bridges over major rivers in rural areas as well as a poor communication system are major obstacles to access (maternal) health services. Similarly, cost of maternal health services including transportation and multiple demands on time of women are also seen to be barriers to affordable maternal health services in Nepal [28].

### Limitation of the study

During the time of the study there was political unrest in Nepal, and it was not safe to visit to some remote areas. There was a political agitation and some people were reluctant to talk to strangers. Data were collected just before the political chance to more democracy took place. Therefore, the study was limited by the areas which where accessible and feasible due to the security reasons which might not comprehensively reflect the normal situation of rural Nepal. Similarly, there were time and resource restrictions, i.e. our study could not cover large areas. The number of households and that of the total population of VDCs was drawn from the last Census and our data were collected by visiting house to house. There was a chance of missing some of the eligible women as the information on which homes had women with children under the age of two came from the FCHVs rather than a recorded population database. The study included targeted women who were at their home, so that there might be selection bias since the visits were during the (working) day. Similarly, due to lack of information and lack of availability about target population we changed our target group to included women having a live child under 24 months rather than 12 months. There might be recall bias arising from this change.

## Conclusion

The study highlighted the poor utilisation of postnatal care in the study area. Only one third of women received a postnatal care. Postnatal care within the most critical period (within 48 hours after delivery) was also very low. It is important to ascertain further why postnatal care rates are so low.

Though barriers to access to postnatal care were thought to be a lack of awareness of postnatal care services, distance to the health facility to visit, poor transportation and roads and lack of trained health workers and postnatal health services in the village, the factors which are found to be associated with postnatal care such as occupation of women and husband, use of antenatal care, use of delivery care and health problems after delivery. These factors probably represent wealth; the value of information motivating postnatal care seeking that is received during antenatal care and the perception that only women with health problems need to make efforts to receive postnatal care.

Health problems during the postnatal period such as weakness, breast infection and vaginal bleeding are not found to be common. The majority who had health problems had sought help and the main care provider for this was the doctor.

### Recommendations

Awareness should be created in the community to motivate pregnant women to attend antenatal care. This will in turn encourage them to seek postnatal care. However, postnatal care services should be made available in the villages, and more health workers and Traditional Birth Attendants should be trained in providing postnatal care. Mothers should be visited at least twice during the postnatal period by local health workers (within 48 hours after delivery and 3–7 days after delivery) as described in the National Maternity Care Guidelines. Awareness programmes on postnatal care should be implemented; targeting women, mother-in-laws and husbands. Moreover, more focus should be on the women who belong to the Tamang group, illiterate and farm-working women and wives of farmers.

## Competing interests

The author(s) declare that they have no competing interests.

## Authors' contributions

SD, GNC, PPS, ERvT and JS have contributed to design the original study including analysis plan and initial interpretation. SD administered the questionnaire in Nepali. All authors assisted in the interpretation of data and provided comments and suggestions on multiple drafted of the papers. All authors read and approved the final submission.

## Pre-publication history

The pre-publication history for this paper can be accessed here:


